# Oedema of the metatarsal heads II-IV and forefoot pain as an unusual manifestation of Lyme disease: a case report

**DOI:** 10.1186/1752-1947-1-44

**Published:** 2007-07-09

**Authors:** Stefan Endres, Markus Quante

**Affiliations:** 1 Department of orthopaedic surgery Elisabeth-Klinik GmbH Bigge/Olsberg, Heinrich-Sommer-Str. 4, 59939 Olsberg, Germany; 2 Department of orthopaedic surgery University of Marburg, Baldingerstrasse, 35039 Marburg, Germany

## Abstract

We report the case of a healthy 36 year old man who suffered from foot pain lasting for weeks, without having a specific medical history relating to it. The clinical evaluation was interpreted as a transfer metatarsalgia caused by a splayfoot. The radiographs revealed no pathology except the splayfoot deformity. Due to persistent pain and swelling of the entire forefoot, after two weeks of conventional treatment, magnet resonance images (MRI) and a blood sample were taken. The laboratory investigation showed raised levels of white blood cell count and C-reactive protein. The MRI showed up oedema in the metatarsal heads II-IV, as well as soft tissue swelling of the forefoot without any signs of decomposition.

Because of this atypical inflammation of the forefoot a laboratory investigation to check for rheumatology disease was done and revealed borrelia burgdorferi infection. On the basis of these findings, antibiotic treatment was started and maintained over three weeks. The symptoms disappeared after 2 weeks, and the patient was able to resume his sports activities.

## Background

Lyme disease has become a relatively common cause of arthritis in areas of the country in which the disease is endemic [1-4]. In the original description of Lyme arthritis, 75% of the patients were children, many of whom were thought by their family physicians to have juvenile rheumatoid arthritis [5]. However, even in adults or adolescents, Lyme arthritis should be diagnosed differently in cases of mono- or oligoarthritis.

The following case highlights an unusual affectation of the forefoot as a result of Borrelia burgdorferi infection.

## Case presentation

A 36 year old man complained of having pain in his left forefoot for 6 weeks. His pain began gradually, unrelated to any specific incident or trauma. The symptoms developed while playing football. He was training for 4 to 8 hours a week. He complained of a sharp, aching pain focused on the metatarsal heads of the left foot. His symptoms had progressed from pain when running to a constant pain that affected his daily living activities. He had swelling and blueish discoloration of the entire forefoot, without any neurologic symptoms. He had never had any previous foot problems, and claimed not to have used new shoes.

After 2 weeks without improvement, nonsteroidal anti-inflammatory medication was prescribed, but he continued to have foot pain. He was not taking any medication except for the NSAIDs, and had no known allergies. Likewise, his family history was unremarkable, and he had a normal social history.

Gait analysis showed mild pronation but no major anomalies. When examined, the affected left forefoot showed persistent swelling and blueish discoloration. Longitudinal arch height was decreased. Compression of the metatarsalia resulted in sharp, aching pain. The talocrural joint had normal plantar flexion, inversion, and eversion. Signs of infection were not evident.

Initial radiographs of the foot were obtained 2 weeks earlier, and the findings were normal. Checking radiographs showed no abnormalities after 2 weeks.

A MRI (magnet resonance images) scan revealed oedema of the metatarsal heads II-IV as well as a soft tissue swelling of the forefoot without any signs of decomposition.

Laboratory investigation showed the following: white blood cell count 14.4 × 10^9^/l, C-reactive protein 21 mg/dl; negative CCP-antibodies, negative antinuclear antibodies and negative HLA-B27. However a positive match of IgM antibodies against Borrelia burgdorferi was found by the post-infectious arthritis laboratory diagnosis.

Treatment was then started with intravenous therapy of ceftriaxone 2 g per day over a period of two weeks, followed by one week of oral therapy of doxycycline 100 mg twice a day.

The symptoms disappeared after two weeks, and the patient was able to return to sports activities after completing the antibiotic treatment.

## Conclusion

The patient in this case had a borrelia burgdorferi infection. The typical annular rash, erythema chronicum migrans (ECM), being characteristic of this disorder was not noticed by the patient, or evident at the first examination by a medical professional. The diagnosis was based on the laboratory diagnostic. Enzyme-linked immunosorbent assay (ELISA) serology and Western blot analysis corroborated a diagnosis of borreliosis. The patient was treated with antiobiotics, and his symptoms improved after a few days.

There are three stages of Lyme disease that have been described: early localised, early disseminated, and late disease.

Early localised disease is seen days to weeks after a tick bite, and is characterized by ECM. Fever, headache, malaise, myalgias, and arthralgias may also be seen.

The early disseminated stage, on the other hand, occurs days to months after a tick bite and can involve many different organ systems. Late Lyme disease is characterised by chronic mono-articular or asymmetric oligo-articular arthritis involving large joints, in particular the knee, but also the smaller joints [6].

The diagnosis of Lyme disease is generally based on clinical presentation. Serologic tests such as ELISA and Western blot analysis may be used to support the clinical diagnosis, but have limited sensitivity and specificity. Polymerase chain reaction (PCR) testing of a skin biopsy from the wound site may detect Borrelia DNA. Treatment options for ECM include two to three weeks of oral amoxicillin and doxycycline [7].

In this special case the diagnosis was delayed because the typical symptoms of Lyme disease were not evident. Atypical pain in the forefoot could be caused by many different diagnoses. The most common cause in adults is a fore foot deformity such as splay foot, especially if the clinical examination and plain radiographs do not reveal other pathologies.

The different diagnosis of persistent metatarsalgia is multifaceted. Morbus Köhler, Morton neurinoma, instability of the metatarsophalangeal joint, claw toes, fractures of the fore foot, tumors, verrucae plantares and arthritis of the metatarsophylangeales (articular gout, rheumatic diseases or infectious arthritis).

In cases of patients with unusual pain such as a metatarsalgia of the fore foot, an algorithm for different diagnoses is useful. First it is necessary to determine if any alteration in the skin can be detected. If there is puckering the diagnosis is almost clear. If not, the next question is whether there are signs of neurological symptoms or signs of arthritis. Neurological symptoms lead to the diagnosis of a Morton neurinoma. Lack of neurological signs and absence of the symptoms of arthritis are mostly associated with instabilities of the metatarsphalangeal joints. Signs of arthritis indicate articular gout, rheumatic or infectious disease, which can be confirmed by serological testing.

## Competing interests

All authors certify they not have signed any agreement with a commercial interest related to this study which would in any way limit publication of any and all data generated for the study or to delay publication for any reason. I confirm that all authors have seen and agree with the contents of the manuscript and agree that the work has not been submitted or published elsewhere in whole or in part. In addition I confirm that patient consent was received for publication of the manuscript and that there are no competing interests.

## Authors' contributions

SE performed the clinical and radiologic evaluation of the patient. MQ participated in the preparation of the manuscript. All authors read and approved the final manuscript.

**Figure 1:**
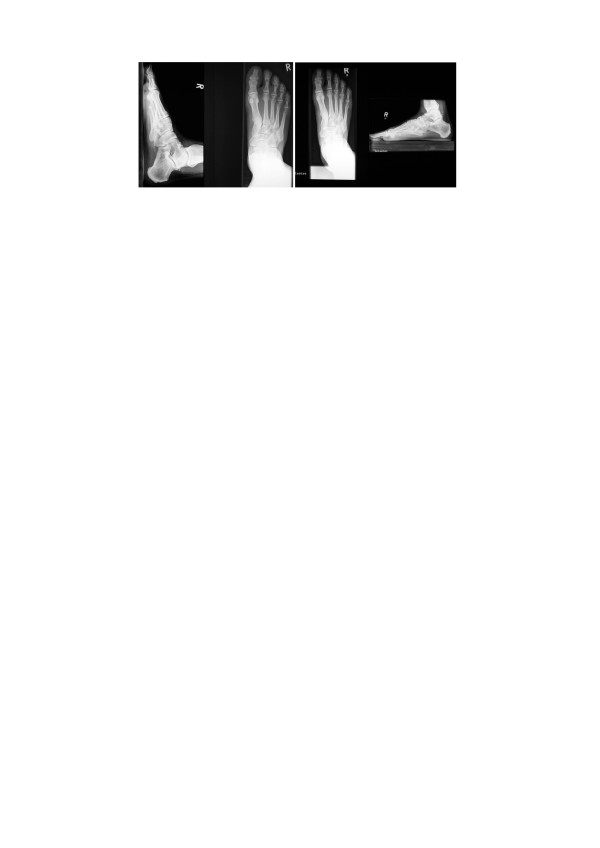
Left – Initial plain radiographs Right – Checking radiographs after 2 weeks.

**Figure 2:**
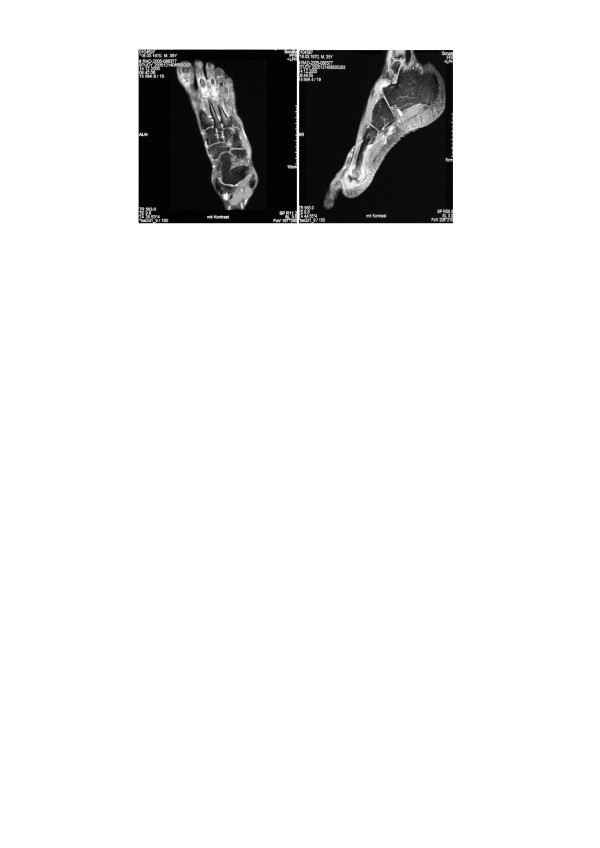
MRI scan of the right foot – oedema of the metatarsal head and soft tissue swelling.
